# Alexithymia, impulsiveness, emotion, and eating dyscontrol: similarities and differences between narcolepsy type 1 and type 2

**DOI:** 10.1007/s41105-022-00414-4

**Published:** 2022-09-05

**Authors:** Chiara Del Bianco, Martina Ulivi, Claudio Liguori, Antonio Pisani, Nicola Biagio Mercuri, Fabio Placidi, Francesca Izzi

**Affiliations:** 1 Sleep Medicine Center, Department of Systems Medicine, Policlinico Tor Vergata, University of Rome “Tor Vergata”, Viale Oxford 81, 00133 Rome, Italy; 2 Neurology Unit, Department of Systems Medicine, University of Rome “Tor Vergata”, Rome, Italy

**Keywords:** Narcolepsy type 1 (NT1), Narcolepsy type 2 (NT2), Impulsiveness, Emotion, Alexithymia, Eating disorder

## Abstract

Non-sleep symptoms, as depression, anxiety and overweight, are often encountered in narcoleptic patients. The purposes of this study are to evaluate mood, impulsiveness, emotion, alexithymia, and eating behavior in patients with narcolepsy type 1 (NT1) and narcolepsy type 2 (NT2) compared to healthy controls and to investigate possible correlations between clinical-demographic data, polysomnographic parameters, and subjective questionnaires. Consecutive patients affected by NT1 and NT2 underwent to Patient Health Questionnaire-9, Generalized Anxiety Disorder-7 Scale, Barratt Impulsivity Scale-11, Difficulties in Emotion Regulation Scale, Toronto Alexithymia Scale, and Eating Disorder Evaluation Questionnaire. Daytime sleepiness was assessed using Epworth sleepiness score. Data were compared with controls. Fourteen NT1, 10 NT2, and 24 healthy subjects were enrolled. Toronto Alexithymia Scale total score was significantly higher in NT1 than NT2. Compared to controls, NT1 patients exhibited significantly higher scores at Patient Health Questionnaire-9 and Difficulties in Emotion Regulation Scale. A positive correlation between hypnagogic hallucinations and Difficulties in emotion regulation was found. NT1 and NT2 share several psycho-emotional aspects, but whereas NT1 patients exhibit more depressive mood and emotion dysregulation compared to controls, alexithymic symptoms are more prominent in NT1 than NT2. Hypnagogic hallucinations, emotion dysregulation, and alexithymia appear to be correlated, supporting the hypothesis of mutual interaction of the above areas in narcolepsy.

## Introduction

Narcolepsy is a chronic debilitating sleep disorder characterized by excessive daytime sleepiness (EDS), sleep paralysis, hypnagogic hallucinations, and fragmented nocturnal sleep. The latest edition of the International Classification of Sleep Disorders (ICSD-3) has classified narcolepsy into narcolepsy type 1 (NT1) and narcolepsy type 2 (NT2) on the basis of levels of hypocretin, also known as orexin, in cerebrospinal fluid (CSF) [1]. Cataplexy is a sudden loss of muscle tone during wakefulness that is evoked by strong, mainly positive, emotions [1]. Patients with NT1, previously reported as narcolepsy with cataplexy, have low levels of hypocretin-1 (HCRT-1) in the CSF, whereas patients with NT2 do not exhibit cataplexy and display normal levels of HCRT-1; however, few patients with narcolepsy and cataplexy with physiological levels of HCRT-1 on CSF have been reported [1]. In addition to sleep/wake-related symptoms, non-sleep disturbances, i.e., depression, anxiety and overweight, are often encountered; the reasons of such relationships possibly stem from the role of hypocretin in several cerebral networks but also from the impact of daytime sleepiness on quality of life. The hypocretins are neuropeptides produced by lateral hypothalamus neurons which widely project to the olfactory bulb, cerebral cortex, thalamus, hypothalamus, brainstem, and limbic system [2, 3]. Therefore, hypocretins play a well-documented role in a variety of physiological processes, such as arousal and the maintenance of wakefulness [4, 5], feeding behavior and energy metabolism [6], neuroendocrine and autonomic functions [7, 8], and emotion regulation and reward processing [9].

Narcolepsy is associated with a high comorbidity of both medical conditions and psychiatric disorders [10–12]. Several studies have reported increased depressive disorders and greater incidence of anxiety disorders in narcolepsy than controls, even in young patients [10, 12, 13], without differences between NT1 and NT2 [11, 14]. A possible genetic link between narcolepsy and depression or others common causal factors still remains speculative [11].

Bayard and coll. observed that NT1 patients opt for choices with higher immediate reward regardless of higher future punishment showing a significant lack of perseverance at the urgency, premeditation, perseverance, sensation seeking (UPPS) Impulsive Behavior scale, compared to healthy subjects [15], whereas data on NT2 are still scarce. An abnormal amygdala function using functional brain magnetic resonance imaging (MRI) has been found in patients with NT1, which may result in abnormal emotional processing under both pleasant and unpleasant conditions [16].

Some authors reported an association of alexithymia in several sleep disorders, including narcolepsy [17]. It has also been assumed that this personality trait may play a role in dream alterations [18] and some authors consider alexithymia a risk factor for somatic symptoms and eating disorders [19]. However, the link between alexithymia and narcolepsy has not yet been well established.

Finally, several studies have shown an increase of body mass index (BMI) and eating concern both in NT1 and NT2 patients, compared with the general population [20], and a high prevalence of eating disorders have been reported in NT1 [21].

Although there is evidence of association between overweight and emotion dysregulation, including alexithymia, in general population [19], their mutual relationships in narcoleptic patients have not been studied so far. Therefore, we performed, for the first time, a comprehensive evaluation of depression, anxiety, impulsiveness, alexithymia, emotion, and eating dysregulation, in narcoleptic patients, comparing NT1 and NT2. Previous studies explored some of these features individually in narcolepsy, but they have not analyzed all these aspects together.

The purposes of this study are (i) to evaluate mood, impulsiveness, emotion, alexithymia, and eating behavior, through self-reported questionnaires, in NT1 and NT2 patients in comparison with healthy controls, and (ii) to investigate possible correlations between clinical and demographic data, polysomnographic findings, excessive daytime sleepiness (EDS), mood, impulsiveness, emotion, alexithymia, and eating behavior in narcoleptic patients.

## Methods

### Participants

NT1 and NT2 patients, diagnosed according to the ICSD-3 criteria, were consecutively enrolled at the Sleep Center of Policlinico Tor Vergata from May 2018 until September 2019. Both treated and untreated narcoleptic patients were included. Age, sex, BMI, disease duration, and medical and psychiatric comorbidities of all participants were collected.

All patients underwent to 24-h Polysomnographic continuous recording (PSG), Multiple Sleep Latency Test (MSLT), and brain MRI. Patients affected by Obstructive Sleep Apnea Syndrome (AHI > 5/h) were excluded. Healthy subjects comparable for age and sex were recruited. Controls were screened by a neurologist with expertise in sleep medicine and subjects with Pittsburg Sleep Quality Index (PSQI) > 5 and/or Epworth Sleepiness Scale (ESS) > 10 were excluded [22]. All subjects gave their written informed consent to the procedures. The study protocol was approved by the Independent Ethical Committee of the University Hospital of Rome “Tor Vergata.”

### Questionnaires

All patients and controls were investigated by means of the following self-administered questionnaires: Patient Health Questionnaire-9 (PHQ-9), Generalized Anxiety Disorder-7 Scale (GAD-7), Barratt Impulsivity Scale-11 (BIS-11), Difficulties in Emotion Regulation Scale (DERS), and Toronto Alexithymia Scale (TAS-20). To assess the presence of obesity and eating disorders we used BMI measurement and Eating Disorder Evaluation Questionnaire 6th edition (EDE-Q). Subjective daytime sleepiness was assessed by ESS [22].

The *Patient Health Questionnaire-9* (PHQ-9) is a brief self-administered nine-item depression-specific questionnaire whose diagnostic validity has been established [23]. The PHQ-9 score ranges from 0 to 27, since each of the 9 items can be scored from 0 (not at all) to 3 (nearly every day). PHQ-9 scores 5 to 9, 10 to 14, 15 to 19, and 20 to 27, correspond to mild, moderate, moderately severe and severe depression, respectively [24].

The *Generalized Anxiety Disorder Scale* (GAD-7) is a seven-item practical self-report anxiety questionnaire that proved valid in primary care and has good operating characteristics for detecting generalized anxiety. The optimal cut point is ≥ 10. Cut points of 5, 10, and 15 might be interpreted as representing mild, moderate, and severe levels of anxiety [25].

The *Barratt Impulsivity Scale-11* (BIS-11) is a self-administered thirty-item questionnaire for measuring impulsiveness trait [26]. The questionnaire comprises six first-order constructs (attention, motor impulsiveness, self-control, cognitive complexity, perseverance, cognitive instability) which in turn form the three second-order factors: Attentional Impulsiveness (IA) refers to the inability to focus on the task at hand, Motor Impulsiveness (IM) involves acting without thinking, and Non-Planning Impulsiveness (NPI) reflects a lack of future orientation. Higher scores indicate higher levels of impulsiveness, with a maximum total score of 120 [26].

The *Difficulties in Emotion Regulation Scale* (DERS) is a thirty-six-item self-reported questionnaire. The sum yields a global DERS score ranging from 36 to 180 with higher scores reflecting greater difficulties in regulating emotion across each domain [27]. The questionnaire comprises of six subscales assessing different dimensions of emotion dysregulation. These subscales include the following: (1) *non-acceptance* of emotion (e.g., “When I’m upset, I become angry with myself for feeling that way”); (2) *Goals* or difficulties engaging in goal-directed behaviors (e.g., “When I’m upset, I have difficulty concentrating”); (3) *Impulse* control difficulties (e.g., “I experience my emotions as overwhelming and out of control”); (4) *Strategies,* as limited access to effective regulatory strategies (e.g., “When I’m upset, I believe that there is nothing I can do to make myself feel better”); (5) *Clarity* as reduced emotional clarity (e.g., “I am confused about how I feel”); and (6) *Awareness* or lack of emotional awareness (e.g., “I pay attention to how I feel” that needs to be coded using a reverse score). A validated Italian version was used [28].

The *Toronto Alexithymia Scale* (TAS-20) is a self-reported questionnaire that comprised twenty items and three factors: (a) difficulty in identifying feelings and differences between feelings and bodily sensations; (b) difficulty in describing feelings; and (c) externally oriented thinking. According to empirically determined cut-off points, people who obtain a score of 61 or more in TAS-20 are identified as being patients with alexithymia [29]. A validated Italian version of TAS-20 was administered [30].

The *Eating Disorder Evaluation Questionnaire 6th edition* (EDE-Q) is a self-report version of the Eating Disorder Examination [31], consisting of thirty six items assessing features of eating disorders and generating four subscale scores: dietary restraint, eating concern, weight concern, and shape concern. Respondents rate the items on a seven-point scale, indicating the number of days out of previous twenty eight in which specific behaviors and attitudes occurred. Higher scores reflect greater degree of severity. The EDE-Q has received psychometric support, including adequate test–retest reliability and a good convergence with the EDE interview [31].

### Statistical analyses

Descriptive statistics are reported as absolute numbers with mean ± standard deviation (SD). Mean item scores were compared between groups using the Student’s *t* test for normally distributed variables. When the normality of the distribution was rejected, as assessed by Kolmogorov–Smirnov test, a non-parametric Kruskal–Wallis test was used for comparisons of median values among three groups, followed by post hoc testing using unpaired Mann–Whitney *U* tests with Bonferroni correction. Statistical significance was set at a *p* < 0.05; after Bonferroni's correction the statistical significance was set at *p* < 0.016. To compare treated and untreated narcoleptic patients, Mann–Whitney *U* test was applied and statistical significance was set at a *p* < 0.05. A non-parametric correlation analysis was conducted through Spearman’s Rho correlation coefficient to assess the associations between the questionnaire scores and PSG and MSLT parameters in the whole sample of narcoleptic patients (NT1 and NT2). To reduce the risk of a Type 1 error, a Bonferroni’s correction indicated a *p* value of 0.008 as acceptable (*p* value of 0.05 divided by the multiple comparisons of PHQ-9; GAD-7, BIS-11, DERS, TAS-20, EDE-Q). Statistical analyses were performed with SPSS Statistics version 20.0.

## Results

### Demographic and clinical data

Twenty-five patients agreed to participate in the study. One patient was excluded for incomplete questionnaires.

Fourteen NT1 patients (8 females and 6 males, mean age 34.78 ± 12.27 y.o.), 10 NT2 (6 females and 4 males, mean age 37.80 ± 15.09 y.o.), and 24 healthy controls (14 females, 10 males, mean age 36.04 ± 13.29 y.o.) were consecutively enrolled at the Sleep Center of Policlinico Tor Vergata from May 2018 until September 2019. NT1 and NT2 did not differ for age, sex, BMI, and disease duration (Table 1). Years of education were significantly higher in controls than NT1 subjects.

**Table 1:** Clinical and demographic characteristics of narcoleptic type 1, type 2 patients, and controls

	NT1 *n* = 14	NT2 *n* = 10	Controls *n* = 24	*p* value	p*	*p*#	*p*°
Sex	8 F, 6 M	6 F, 4 M	14 F, 10 M	0.99	0.89	0.94	0.92
Age, *y*	34.78 ± 12.27	37.80 ± 15.09	36.04 ± 13.29	0.88	0.73	0.66	0.82
Education, *y*	11.71 ± 2.36	13.00 ± 2.40	13.95 ± 1.51	**0.006**	0.09	**0.001**	0.36
Disease duration,*y*	12.47 ± 7.71	13.30 ± 9.03	n.a	–	0.97	–	–
ESS	12.28 ± 5.63	13.30 ± 5.55	5.16 ± 3.65	** < 0.001**	0.55	** < 0.001**	** < 0.001**
BMI	26.84 ± 6.55	26.28 ± 1.84	23.58 ± 3.72	0.078	0.70	0.12	0.03

Data are expressed as mean ± SD

*NT1* Narcolepsy type1, *NT2* Narcolepsy type 2, *ESS* Epworth sleepiness scale, *BMI* Body mass index, *n.a.* Not applicable

*p* value, comparison among NT1, NT2, and controls by means of Kruskal–Wallis test p* comparison between NT1 and NT2

*p*# comparison between NT1 and controls

*p*° comparison between NT2 and controls using Mann–Whitney test. Bold values denote statistical significance

All NT1 and NT2 patients complained Excessive Daytime Sleepiness (EDS), sleep paralysis was reported by 6 NT1 and 6 NT2 patients (50%), hypnagogic hallucinations by 7 NT1 and 3 NT2 patients (41%). All NT1 patients reported cataplexy. As expected, the ESS score was significantly higher in NT1 and NT2 patients than controls (*p* = 0.001), while it did not differ between NT1 and NT2 (Table 1).

HLA study was available for 13 patients (9 NT1 and 4 NT2) and 11 narcoleptic patients were positive for HLA DQB1*0602 (9 NT1 and 2 NT2). CSF HCRT-1 level was available in seven out of 13 NT1 subjects (mean 35.33 ± 20.42 pg/ml, range 10–81.5) and in 2 NT2 (126 and 214 pg/ml, respectively), both positive for HLA DQB1* 0602.

PSG recordings were performed in all patients; mean total sleep time was comparable in NT1 and NT2 subjects (345.69 ± 54.78 vs 373.11 ± 91.05, min, *p* = 0.17), sleep efficiency (SE) was significantly higher in NT2 than NT1 (92.25 ± 4.22 vs 80.58 ± 8.04, %, *p* = 0.001) and wakefulness after sleep onset (WASO) was significantly higher in NT1 than NT2 (68.15 ± 41.93 min vs 22 ± 16.66, *p* = 0.007). No statistical differences were found between NT1 and NT2 as regard to sleep onset (16.53 ± 18.83 vs 6.33 ± 6.76, *p* = 0.44) and percentages of stage N1 (9.84 ± 7.34 vs 8.05 ± 6.29, *p* = 0.75), of stage N2 (46.72 ± 10.82 vs 47.48 ± 5.41, *p* = 0.39), of stage N3 (23.96 ± 10.52 vs 23.57 ± 7.08, *p* = 0.88) and REM sleep (19.47 ± 6.20 versus 20.97 ± 5.20, *p* = 0.52).

At MSLT, mean sleep latencies were comparable in NT1 and NT2 (5.21 ± 4.1 vs 4.1 ± 2.1 min; *p* = 0.73) as well as the number of Sleep Onset REM Periods (SOREMPs) (2.42 ± 1.34 vs 1.55 ± 0.88; *p* = 0.12).

Brain MRI was unremarkable in all patients.

Three NT1 patients had psychiatric comorbidities as depression, 1 NT2 patient had hypothyroidism and migraine, 2 NT1 had only migraine, and 1 NT2 had ulcerative colitis.

Finally, 58% of 24 patients were under treatment. More in detail, 5 NT1 and 5 NT2 patients were drug naïve, whereas 9 NT1 and 5 NT2 were on pharmacological treatment (3 NT2 on modafinil as monotherapy, 3 NT1 and 2 NT2 on pitolisant as monotherapy, 5 NT1 on pitolisant as add-on to modafinil, and 1 NT1 sodium oxybate as add-on to modafinil). ESS score did not differ between treated and drug-naïve patients (11.70 ± 5.16 vs 13.42 ± 5.81, *p* = 0.35).

### Questionnaires

#### Depression and anxiety

Patients affected by NT1 showed a significantly higher PHQ-9 global score than controls (*p* = 0.001). Ten out of 24 patients exhibited a PHQ-9 score > 10 (7 NT1 and 2 NT2) and one NT2 patient showed PHQ-9 score > 20 indicating moderate and severe degree of depression, respectively.

Mean GAD-7 global score was slightly higher in NT1 and NT2 patients than controls, without reaching statistical significance. GAD-7 score was > 10 (moderate anxiety) in one NT1 patient and > 15 (severe anxiety) in three out of 24 narcoleptic subjects (1 NT1 and 2 NT2).

No statistical differences were found in the comparison between NT1 and NT2 at PHQ-9 and GAD-7 questionnaires. Data are reported in Table 2.

**Table 2:** Questionnaires: comparison between patients with narcolepsy type 1, type 2, and controls

	NT1 *n* = 14	NT2 *n* = 10	Controls *n* = 24	*p* value	p*	*p*#	*p*°
PHQ-9	9.78 ± 3.70	9.60 ± 7.19	5.20 ± 3.28	**0.005**	0.59	**0.001**	0.08
GAD-7	5.85 ± 3.46	7.00 ± 7.02	5.41 ± 3.42	0.90	0.74	0.67	0.94
BIS-11 total score	58.85 ± 9.23	54.40 ± 8.65	56.75 ± 8.23	0.62	0.36	0.57	0.54
Attentional impulsiveness	15.07 ± 2.36	14.90 ± 3.03	14.54 ± 3.56	0.66	0.88	0.37	0.63
Motor impulsiveness	20.14 ± 4.65	18.40 ± 3.33	18.29 ± 2.92	0.57	0.42	0.32	0.91
Non-planning impulsiveness	23.78 ± 6.55	21.10 ± 6.47	23.91 ± 3.45	0.57	0.63	0.54	0.32
DERS total score	72.15 ± 19.85	61.90 ± 24.52	56.12 ± 9.49	0.04	0.14	**0.009**	0.57
Non-acceptance	12.92 ± 6.60	12.30 ± 8.11	9.33 ± 3.22	0.32	0.55	0.10	0.70
Goals	13.07 ± 5.43	12.20 ± 7.80	9.29 ± 2.44	0.16	0.41	0.038	0.81
Strategies	17.00 ± 5.18	15.90 ± 6.91	13.04 ± 3.60	0.08	0.51	0.018	0.42
Impulse	11.61 ± 5.88	8.50 ± 3.43	8.00 ± 2.34	0.14	0.18	0.053	0.92
Awareness	10.38 ± 3.04	7.90 ± 2.51	9.25 ± 2.28	0.14	0.07	0.24	0.19
Clarity	6.92 ± 2.98	5.10 ± 2.55	7.20 ± 2.18	0.07	0.14	0.65	0.019
TAS-20 total score	49.07 ± 10.85	36.70 ± 5.25	40.62 ± 9.01	**0.010**	**0.005**	0.018	0.33
Difficulty in identifying feelings	16.07 ± 6.79	10.40 ± 5.05	11.16 ± 4.09	0.024	0.017	0.026	0.34
Difficulty in describing feelings	13.64 ± 3.85	9.20 ± 2.39	12.08 ± 4.26	0.020	**0.004**	0.17	0.08
Externally oriented thinking	19.35 ± 5.25	17.10 ± 5.54	16.83 ± 4.80	0.30	0.33	0.12	0.77
EDE-Q total score	6.99 ± 4.61	5.53 ± 4.31	4.04 ± 3.92	0.10	0.52	0.38	0.27
Dietary restraint	1.37 ± 1.41	1.88 ± 1.37	0.89 ± 0.99	0.05	0.15	0.25	0.018
Eating concern	1.08 ± 0.90	0.68 ± 1.13	0.62 ± 0.96	0.055	0.08	0.022	0.95
Shape concern	2.51 ± 1.70	2.09 ± 1.77	1.49 ± 1.40	0.17	0.59	0.049	0.47
Weight concern	2.02 ± 1.38	1.58 ± 1.53	1.03 ± 1.10	0.08	0.53	0.025	0.27

Data are expressed as mean ± SD

*NT1* Narcolepsy type 1, *NT2* Narcolepsy type 2, *PHQ-9* Patient Health Questionnaire-9, *GAD-7* Generalized Anxiety Disorder-7, *BIS-11* Scale Barratt Impulsivity Scale-11; *DERS* Difficulties in Emotion Regulation Scale, *TAS-20* Toronto Alexithymia Scale; *EDE-Q* Eating Disorder Evaluation Questionnaire

*p* value, comparison among NT1, NT2, and controls by means of Kruskal–Wallis test; p* comparison between NT1 and NT2

*p*# comparison between NT1 and controls

*p*° comparison between NT2 and controls using Mann–Whitney test

Bold values denote statistical significance. Statistical significance was set at *p* < 0.016

#### Impulsivity

BIS-11 total score and the subscales scores did not differ among NT1, NT2, and healthy controls (Table 2).

#### Emotion dysregulation

DERS global score was significantly higher in NT1 patients compared to controls (*p* = 0.009) (Table 2).

Compared to healthy subjects, the scores at “Goals” and “Strategies” sub-items were higher in NT1 patients and “Clarity” sub-item was higher in NT2 patients, but without reaching the statistical significance after Bonferroni's correction (Table 2).

#### Alexithymia

TAS-20 global score was significantly higher in NT1 than in NT2 (*p* = 0.005) (Table2).

Furthermore, the “difficulty in identifying feelings” (DIF) and the “difficulty in describing feeling” (DDF) subscale scores were higher in NT1 than in NT2 patients (*p* = 0.017 and *p* = 0.004), without reaching the statistical significance, after Bonferroni's correction as to TAS-DIF (Table 2).

A TAS-20 score ≥ 61, suggestive of alexithymia, was observed in 3 out of 14 NT1 patients, whereas all controls and NT2 exhibited TAS-20 score < 61.

#### Eating disorders

EDE-Q total score did not differ between NT1, NT2, and controls.

Although “dietary restrain” score was higher in NT2 than controls (*p* = 0.018) and “eating concern,” “shape concern,” and “weight concern” sub-items scores were higher in NT1 than in healthy subjects, these differences lost the statistical significance after Bonferroni's correction (Table2). No statistical differences emerged from the comparison between NT1 and NT2 (Table 2).

#### Comparison between treated and drug-naïve patients

No statistical differences emerged in the questionnaires (PHQ-9, GAD-7, BIS-11, DERS, TAS-20, and EDE-Q) in the comparison between patients on pharmacological treatment (9 NT1 and 5 NT2) and drug-naïve patients (5 NT1 and 5 NT2).

#### Correlations between clinical, demographic, PSG data, and subjective questionnaires in narcoleptic patients

No correlation among age, age at narcolepsy onset, disease duration and PSG data, and Questionnaires scores were found in the whole sample of narcoleptic patients (NT1 and NT2).

Correlations between PSG data and subjective questionnaires, as ESS and PHQ-9 correlation, lost statistical significance after Bonferroni correction.

Hypnagogic hallucinations was positively associated with DERS total score (*p* < 0.001), Goals (*p* < 0.001), Impulse (*p* = 0.002), and Strategies (*p* < 0.001). The positive correlation with DERS NA (*p* = 0.023) and TAS-DIF (*p* = 0.02) lost the statistical significance after Bonferroni's correction.

All data are reported in Table 3.

**Table 3:** Spearman’s rank correlation between clinical, demographic data, PSG and MSLT parameters, and questionnaires in narcoleptic patients type 1 and type 2

		Age	Age at onset	Disease duration	Education	BMI	ESS	TST	SE	Sleep onset	WASO	N1	N2	N3	REM	REM latency	MSL	SOREMPs	Hyp hal
PHQ-9	*r*	− .136	− .126	− .182	.010	.006	.423^*^	− .071	− .077	− .036	.053	.189	− .184	.055	− .305	.139	− .016	.105	.502
	*p*	.527	.559	.394	.961	.977	.039	.752	.733	.874	.815	.413	.425	.813	.178	.537	.943	.633	.012
GAD-7	*r*	.168	.007	.176	− .117	− .086	.097	− .411	− .112	.126	− .136	.486^*^	− .112	− .236	− .233	.272	.093	− .100	.349
	*p*	.433	.973	.410	.587	.690	.652	.058	.619	.575	.545	.026	.628	.303	.310	.221	.675	.649	.095
BIS total	*r*	.342	.267	.090	− .329	− .002	− .338	− .080	− .441^*^	.143	.484^*^	.071	.072	− .177	.124	.030	− .297	.151	.135
	*p*	.102	.207	.677	.116	.991	.107	.722	.040	.525	.023	.761	.755	.442	.591	.894	.169	.491	.530
AI	*r*	− .094	− .196	.157	− .212	− .068	− .086	− .404	− .105	− .127	.069	.177	− .468^*^	.362	− .254	.023	.060	.037	− .074
	*p*	.663	.359	.464	.321	.752	.689	.062	.640	.572	.761	.443	.032	.107	.267	.921	.786	.866	.732
MI	*r*	.129	.246	− .251	− .253	.016	− .121	− .159	− .406	.062	.382	.242	.200	− .267	− .165	.046	− .235	− .063	.141
	*p*	.548	.246	.236	.232	.941	.574	.481	.061	.783	.079	.291	.384	.241	.475	.840	.280	.775	.511
NPI	*r*	.294	.246	.101	− .069	− .006	− .400	.182	− .263	.190	.357	− .128	.118	− .108	.231	.119	− .267	.230	.153
	*p*	.164	.246	.639	.750	.977	.053	.418	.237	.396	.103	.580	.610	.641	.313	.598	.218	.290	.475
DERS total	*r*	− .144	− .272	.038	.052	− .047	.321	− .406	− .310	.148	.034	.083	− .045	− .028	− .336	.057	− .173	.291	.721**
	*p*	.513	.209	.862	.813	.830	.135	.068	.171	.521	.882	.728	.851	.906	.147	.806	.442	.189	**.000**
NA	*r*	.184	− .047	.223	.167	.120	.126	− .263	− .262	.308	− .061	.286	.080	− .303	− .252	.146	− .379	.307	.472
	*p*	.401	.831	.306	.446	.587	.566	.249	.251	.174	.794	.221	.736	.194	.284	.527	.082	.164	.023
Goals	*r*	− .120	− .251	− .013	.250	− .041	.403	− .128	− .144	.176	− .046	.173	− .180	− .067	− .181	.176	− .223	.285	.670**
	*p*	.586	.248	.952	.250	.854	.057	.580	.532	.445	.843	.466	.446	.780	.446	.445	.319	.199	**.000**
Strategies	*r*	− .077	− .256	.178	.168	− .070	.330	− .435^*^	− .177	.015	.011	.069	.028	.019	− .401	.203	− .180	.287	.698**
	*p*	.728	.238	.417	.444	.752	.125	.049	.443	.949	.962	.772	.907	.938	.080	.376	.424	.196	**.000**
Impulse	*r*	− .030	.090	− .252	− .006	− .046	.258	− .008	− .314	.027	.354	.332	− .289	− .023	− .261	.023	− .335	.292	.621**
	*p*	.892	.682	.246	.977	.833	.235	.973	.166	.908	.115	.153	.217	.924	.266	.920	.127	.187	**.002**
Awareness	*r*	− .134	− .102	− .011	− .169	− .247	− .217	− .231	− .378	.064	.290	.219	− .041	− .090	− .169	.009	.057	.092	.393
	*p*	.543	.644	.959	.440	.256	.320	.313	.091	.782	.203	.354	.864	.706	.475	.971	.801	.682	.064
Clarity	*r*	− .186	− .192	.022	− .391	− .154	.096	− .337	− .091	− .171	− .029	− .269	.073	.015	.119	− .279	.299	− .165	.047
	*p*	.396	.379	.919	.065	.482	.663	.135	.696	.459	.902	.252	.760	.949	.618	.221	.176	.463	.832
TAS-20 total	*r*	.064	− .158	.179	− .492^*^	.304	− .091	− .376	− .483^*^	− .095	.384	− .085	− .014	.091	− .146	− .143	.214	.109	.073
	*p*	.767	.461	.404	.015	.149	.674	.084	.023	.674	.078	.715	.952	.693	.526	.525	.327	.621	.733
DIF	*r*	.211	− .074	.225	− .394	.090	.157	− .415	− .309	− .059	.256	.234	− .202	.013	− .201	− .063	.128	.188	.473*
	*p*	.323	.732	.291	.057	.676	.464	.055	.162	.793	.250	.308	.380	.955	.382	.779	.560	.392	.020
DDF	*r*	− .092	− .338	.204	− .151	.217	− .047	− .194	− .452^*^	.056	.271	− .064	− .088	.025	− .151	.016	.174	.333	.080
	*p*	.670	.106	.340	.481	.309	.828	.388	.035	.804	.222	.783	.705	.916	.514	.945	.428	.121	.711
EOT	*r*	− .035	.096	− .040	− .406^*^	.328	− .188	− .176	− .342	− .007	.218	− .172	.307	− .093	− .012	− .056	.280	− .162	− .398
	*p*	.869	.655	.854	.049	.118	.379	.434	.119	.977	.329	.456	.176	.689	.960	.804	.195	.460	.054
EDE − Q total	*r*	.253	.017	.173	− .212	.200	.068	.053	− .137	.253	− .055	.086	.149	− .294	.032	.413	.155	.037	.098
	*p*	.232	.937	.418	.321	.350	.754	.816	.542	.256	.809	.712	.520	.195	.889	.056	.481	.867	.650
DR	*r*	.445^*^	.259	.268	− .004	.134	.179	.250	.312	.018	− .371	.266	.459^*^	− .552^**^	− .007	.255	.181	− .138	.117
	*p*	.030	.221	.205	.985	.531	.402	.261	.158	.936	.089	.244	.037	.009	.975	.252	.409	.529	.588
EC	*r*	.083	.008	.012	− .236	.274	− .041	.102	− .427^*^	.215	.292	.216	− .014	− .274	− .095	.190	.129	.134	.191
	*p*	.700	.971	.957	.267	.194	.849	.651	.047	.336	.187	.347	.952	.229	.684	.398	.557	.541	.371
SC	*r*	.066	− .038	− .080	− .133	.133	.247	.114	− .081	.210	− .118	− .060	.220	− .265	.035	.276	.033	− .002	.208
	*p*	.760	.861	.709	.537	.535	.245	.613	.718	.349	.601	.796	.339	.246	.880	.214	.881	.993	.330
WC	*r*	.085	− .091	− .002	− .138	.078	.120	.160	− .036	.255	− .121	− .011	.183	− .248	− .008	.342	.097	.047	.233
	*p*	.692	.671	.994	.521	.718	.575	.477	.872	.253	.591	.963	.426	.279	.971	.119	.659	.830	.273

Age, age at onset, disease duration, and education are expressed in years

PSG parameters: *TST* Total Sleep time in minutes, *SE* Sleep efficiency in percentage; sleep onset in minutes WASO, wake after sleep onset in minutes; N1, N2, N3 stages and REM sleep in percentage; REM latency in minutes. MSLT data: *MSL* mean sleep latency in minutes, *SOREMPs* number of sleep onset of REM periods. *Hyp Hal* Hypnagogic hallucinations. *PHQ-9* Patient Health Questionnaire-9, *GAD-7* Generalized anxiety disorder-7 scale, *BIS-11* Barratt Impulsivity Scale-11; BIS subscales (*AI* Attentional Impulsiveness, *MI* Motor impulsiveness, *NP* Non-planning Impulsiveness); *DERS* Difficulties in Emotion Regulation Scale; DERS subscales (*NA* non-acceptance). *TAS-20* Toronto Alexithymia Scale-20; TAS subscales (*DIF* Difficulty in identifying feelings, *DDF* Difficulty in describing feelings, *EOT* Externally oriented thinking). *EDE-Q* Eating Disorder Evaluation Questionnaire; EDE-Q subscales (*DR* Dietary restraint, *EC* Eating concern, *SC* Shape concern, *WC* Weight concern). *r* Spearman’s rho correlation coefficient

^*^p < 0.05, in light gray, denotes weak correlation

^**^p < 0.01, in dark gray, denotes strong correlation

Bold values denote statistical significance after Bonferroni’s correction (*p* value < 0.008)

#### Correlations between subjective questionnaires in narcoleptic patients

PHQ-9 total score showed positive correlations with GAD-7 (*p* = 0.002), DERS total score (*p* < 0.001), Goals (*p* < 0.001), Strategy and Impulse (*p* < 0.001), and TAS-DIF (*p* = 0.006).

GAD-7 was also positively associated with DERS total score (*p* = 0.002), and TAS-DIF (*p* = 0.003).

DERS total score was positively associated with TAS-DIF (*p* < 0.001), while DERS Goals was associated with TAS- EOT (*p* = 0.003). TAS-DIF was positively associated with DERS total score (*p* < 0.001).

Correlations between questionnaires total scores and their subscales were excluded.

All data are reported in Table 4.

**Table 4:** Spearman’s rank correlation between subjective questionnaires in narcoleptic patients type 1 and type 2

		PHQ-9	GAD7	BIS tot	AI	MI	NPI	DERS tot	NA	Goals	Strategies	Impulse	Awareness	Clarity	TAS-20 tot	DIF	DDF	EOT	EDE-Q Tot	DR	EC	SC	WC
PHQ-9	*r*	1.000	.588^**^	− .043	.424^*^	. 259	− .344	.733^**^	.338	.758^**^	.674^**^	.772^**^	.227	− .105	.232	.543^**^	.207	− .231	.349	− .104	.427^*^	.528^**^	.465^*^
	*p*		**.002**	.842	.039	.221	.099	**.000**	.115	**.000**	**.000**	**.000**	.298	.633	.276	**.006**	.332	.277	.094	.629	.037	.008	.022
GAD-7	*r*	.588^**^	1.000	.103	.480^*^	.376	− .331	.604^**^	.526^**^	.468^*^	.512^*^	.413	.352	.141	.278	.580^**^	.102	− .114	.478^*^	.294	.383	.466^*^	.511^*^
	*p*	**.002**		.634	.018	.071	.114	**.002**	.010	.024	.013	.050	.099	.522	.188	**.003**	.634	.597	.018	.163	.065	.022	.011
BIS total	*r*	− .043	.103	1.000	.131	.801^**^	.777^**^	.098	.227	.035	.014	.299	.100	.055	.373	.294	.300	.187	.274	− .119	.371	.180	.218
	*p*	.842	.634					.656	.299	.875	.948	.166	.649	.801	.072	.163	.155	.382	.196	.579	.074	.400	.306
AI	*r*	.424^*^	.480^*^	.131	1.000	.208	− .364	.286	.117	.304	.231	.272	− .147	.159	.261	.387	.237	− .073	.019	− .349	.048	− .020	− .025
	*p*	.039	.018					.185	.595	.158	.288	.209	.503	.467	.218	.061	.265	.734	.930	.094	.824	.926	.909
MI	*r*	.259	.376	.801^**^	.208	1.000	.396	.318	.390	.225	.145	.538^**^	.172	− .005	.430^*^	.372	.308	.208	.364	− .032	.506^*^	.394	.384
	*p*	.221	.071					.140	.066	.301	.508	.008	.434	.982	.036	.073	.143	.330	.080	.884	.012	.056	.064
NPI	*r*	− .344	− .331	.777^**^	− .364	.396	1.000	− .142	.042	− .126	− .136	.077	.104	− .073	.042	− .072	.115	.085	.095	− .074	.140	.004	.072
	*p*	.099	.114					.519	.850	.566	.536	.726	.637	.741	.846	.739	.594	.692	.660	.729	.514	.984	.736
DERS total	*r*	.733^**^	.604^**^	.098	.286	.318	− .142	1.000	.669^**^	.824^**^	.910^**^	.689^**^	.453^*^	.224	.421^*^	.703^**^	.440^*^	− .217	.183	− .192	.305	.329	.303
	*p*	**.000**	**.002**	.656	.185	.140	.519								.046	**.000**	.036	.319	.402	.381	.158	.125	.160
NA	*r*	.338	.526^**^	.227	.117	.390	.042	.669^**^	1.000	.670^**^	.537^**^	.491^*^	.042	− .207	.218	.352	.374	− .258	.280	.109	.321	.301	.358
	*p*	.115	.010	.299	.595	.066	.850								.317	.099	.079	.235	.196	.621	.136	.164	.093
Goals	*r*	.758^**^	.468^*^	.035	.304	.225	− .126	.824^**^	.670^**^	1.000	.682^**^	.743^**^	.081	− .161	.087	.509^*^	.412	− 588^**^	.225	− .159	.315	.352	.356
	*p*	**.000**	.024	.875	.158	.301	.566								.694	.013	.051	**.003**	.303	.470	.143	.099	.096
Strategies	*r*	.674^**^	.512^*^	.014	.231	.145	− .136	.910^**^	.537^**^	.682^**^	1.000	.535^**^	.438^*^	.223	.343	.633^**^	.266	− .201	.064	− .173	.092	.183	.157
	*p*	**.000**	.013	.948	.288	.508	.536								.110	**.001**	.220	.357	.773	.429	.678	.404	.475
Impulse	*r*	.772^**^	.413	.299	.272	.538^**^	.077	.689^**^	.491^*^	.743^**^	.535^**^	1.000	.297	− .234	.161	.471^*^	.282	− .323	.226	− .272	.494^*^	.383	.334
	*p*	**.000**	.050	.166	.209	.008	.726								.464	.023	.193	.133	.300	.209	.017	.071	.119
Awareness	*r*	.227	.352	.100	− .147	.172	.104	.453^*^	.042	.081	.438^*^	.297	1.000	.395	.369	.462^*^	.168	.056	.033	− .074	.200	.107	.131
	*p*	.298	.099	.649	.503	.434	.637								.083	.027	.444	.799	.880	.738	.361	.626	.552
Clarity	*r*	− .105	.141	.055	.159	− .005	− .073	.224	− .207	− .161	.223	− .234	.395	1.000	.507^*^	.423^*^	.231	.444^*^	− .135	− .114	− .227	− .144	− .155
	*p*	.633	.522	.801	.467	.982	.741								.014	.044	.290	.034	.540	.605	.298	.512	.480
TAS-20 total	*r*	.232	.278	.373	.261	.430^*^	.042	.421^*^	.218	.087	.343	.161	.369	.507^*^	1.000	.754^**^	.750^**^	.520^**^	.313	− .132	.407^*^	.264	.256
	*p*	.276	.188	.072	.218	.036	.846	.046	.317	.694	.110	.464	.083	.014					.136	.540	.048	.213	.228
DIF	*r*	.543^**^	.580^**^	.294	.387	.372	− .072	.703^**^	.352	.509^*^	.633^**^	.471^*^	.462^*^	.423^*^	.754^**^	1.000	.585^**^	− .023	.276	− .073	.318	.242	.276
	*p*	**.006**	**.003**	.163	.061	.073	.739	**.000**	.099	.013	**.001**	.023	.027	.044					.192	.733	.131	.255	.191
DDF	*r*	.207	.102	.300	.237	.308	.115	.440^*^	.374	.412	.266	.282	.168	.231	.750^**^	.585^**^	1.000	.125	.258	− .194	.452^*^	.225	.249
	*p*	.332	.634	.155	.265	.143	.594	.036	.079	.051	.220	.193	.444	.290					.224	.364	.027	.291	.240
EOT	*r*	− .231	− .114	.187	− .073	.208	.085	− .217	− .258	− 588^**^	− .201	− .323	.056	.444^*^	.520^**^	− .023	.125	1.000	.172	.073	.197	.111	.036
	*p*	.277	.597	.382	.734	.330	.692	.319	.235	**.003**	.357	.133	.799	.034					.422	.734	.356	.606	.867
EDE-Q total	*r*	.349	.478^*^	.274	.019	.364	.095	.183	.280	.225	.064	.226	.033	− .135	.313	.276	.258	.172	1.000	.591^**^	.755^**^	.922^**^	.947^**^
	*p*	.094	.018	.196	.930	.080	.660	.402	.196	.303	.773	.300	.880	.540	.136	.192	.224	.422					
DR	*r*	− .104	.294	− .119	− .349	− .032	− .074	− .192	.109	− .159	− .173	− .272	− .074	− .114	− .132	− .073	− .194	.073	.591^**^	1.000	.278	.462^*^	.535^**^
	*p*	.629	.163	.579	.094	.884	.729	.381	.621	.470	.429	.209	.738	.605	.540	.733	.364	.734					
EC	*r*	.427^*^	.383	.371	.048	.506^*^	.140	.305	.321	.315	.092	.494^*^	.200	− .227	.407^*^	.318	.452^*^	.197	.755^**^	.278	1.000	.731^**^	.742^**^
	*p*	.037	.065	.074	.824	.012	.514	.158	.136	.143	.678	.017	.361	.298	.048	.131	.027	.356					
SC	*r*	.528^**^	.466^*^	.180	− .020	.394	.004	.329	.301	.352	.183	.383	.107	− .144	.264	.242	.225	.111	.922^**^	.462^*^	.731^**^	1.000	.957^**^
	*p*	.008	.022	.400	.926	.056	.984	.125	.164	.099	.404	.071	.626	.512	.213	.255	.291	.606					
WC	*r*	.465^*^	.511^*^	.218	− .025	.384	.072	.303	.358	.356	.157	.334	.131	− .155	.256	.276	.249	.036	.947^**^	.535^**^	.742^**^	.957^**^	1.000
	*p*	.022	.011	.306	.909	.064	.736	.160	.093	.096	.475	.119	.552	.480	.228	.191	.240	.867					

*PHQ-9* Patient Health Questionnaire-9, *GAD-7* Generalized Anxiety Disorder-7 Scale, *BIS-11* Barratt Impulsivity Scale-11; BIS subscales (*AI* Attentional impulsiveness, *MI* Motor impulsiveness, *NP* Non-planning impulsiveness), *DERS* Difficulties in Emotion Regulation Scale, *DERS* Subscales (*NA* Non-acceptance). *TAS-20* Toronto Alexithymia Scale-20, TAS subscales (*DIF* Difficulty in identifying feelings, *DDF* Difficulty in describing feelings, *EOT* Externally oriented thinking). *EDE-Q* Eating Disorder Evaluation Questionnaire, EDE-Q subscales (*DR* Dietary restraint, *EC* Eating concern, *SC* Shape concern, *WC* Weight concern); *r* Spearman’s rho correlation coefficient

^*^p < 0.05 in light gray, denotes weak correlation

^**^p < 0.01 in dark gray, denotes strong correlation

Bold values denote statistical significance after Bonferroni’s correction (*p* value < 0.008)

## Discussion

The peculiarity of our study is the comprehensive evaluation of the psycho-emotional profile investigating the mutual relationships between mood, emotion, eating behavior, alexithymic trait, impulsiveness, daytime somnolence, and PSG parameters in a limited but representative sample of NT1 and NT2 patients compared to healthy control group.

An association between emotion dysregulation and sleep disorder has been described and difficulties in modulating emotional experiences, evaluated through DERS questionnaire, have been observed in patients suffering from insomnia [32]. For the first time the DERS questionnaire was administered in patients with narcolepsy.

We found a statistically higher DERS global score in NT1 patients than controls. The impairment in emotion processing and the possible occurrence of different emotional networks in NT1 are still debated [33–35]. The hypothalamus, including the hypocretin region in close anatomical connection with the amygdala, is crucial in human emotional processing [5]; therefore, it is expected that the dysfunction of hypocretin system, which is pathognomonic of NT1, may impair the above circuit. Considering the crucial role of emotions in cataplexy, several authors have hypothesized that NT1 could experience an emotional constriction to avoid cataplexy attacks [33, 34]. Furthermore, in line with this hypothesis, in our sample NT1 patients exhibited higher difficulty in identifying and describing feelings at alexithymia scale than NT2. On the matter, findings of previous studies are controversial. It was reported that emotional judgment ability, evaluated by a classical emotional processing task (i.e., facial affect recognition) and explicit emotion regulation strategies detected through a self-reported questionnaire (Emotional Regulation Questionnaire), is comparable in NT1, NT2, and controls [35]. Moreover, since REM sleep and dreaming play a role in the cross-night regulation of negative emotions, it has been hypothesized that alexithymia may be related to dream alterations in NT1 [36]. In this regard, alexithymia is positively related with nightmare distress in other sleep disturbances [37, 38].

We found a positive correlation between hypnagogic hallucinations and DERS total score, while the same correlation with TAS-DIF (*p* = 0.02) lost the statistical significance after Bonferroni's correction.

Our findings, for the first time, support the hypothesis that narcoleptic patients show an impairment in emotional processing, evaluated by means of DERS, and that emotion dysregulation is correlated with the presence of hypnagogic hallucinations.

In a previous study, an association between nightmare distress and the DIF component of alexithymia was described, both for patients with sleep disorders and for healthy subjects, and it has been assumed that both nightmare distress and DIF scores reflect a deficit in the processes of regulating emotions [17].

Emotion dysregulation may lead alexithymic individuals to exhibit excessive emotional outbursts when they are awake [17, 37]. As above explained, dreaming and REM sleep play role in the cross-night regulation of negative emotions and the consolidation of emotional memories. In our study the positive correlation between hypnagogic hallucinations (associated with nightmares and REM sleep) and DERS supports the hypothesis of a mutual interaction between REM alteration and dysregulation of diurnal emotions, in narcoleptic patients.

Narcoleptic subjects, in particular NT1, exhibit higher score than controls in the depression-specific questionnaire (PHQ-9) without statistical differences between NT1 and NT2, in line with previous studies [11]. Nevertheless, no differences in anxiety-specific questionnaire (GAD-7) score between narcoleptic patients and controls were found in our population, in contrast with other studies describing augmented anxiety disorder in narcolepsy [10, 14]. However, our findings are not fully comparable to previous data since different methods were utilized and because the distinction between NT1 and NT2 was not always performed in above-mentioned studies.

We found a positive correlation between PHQ-9 total score and GAD-7, PHQ-9 and DERS total score, and PHQ-9 and TAS-DIF and also between GAD-7 and DERS total score. DERS total score and TAS-DIF are also positively associated with each other.

These findings support the hypothesis of mutual interaction between depression, anxiety, emotion dysregulation, and alexithymia in narcoleptic patients.

Regarding eating dyscontrol, in animal models the dysfunction in reward circuits may contribute to eating disorders (anorexia, bulimia, and binge eating) [39] and some authors observed in NT1 patients higher scores on the Eating Attitude Test [20] and various types of eating disorders, including Eating Disorder Not Otherwise Specified, with increased weight concern and binge eating [9, 20]. In addition, impulsivity may be a characteristic of people who exhibit aspects of abnormal eating and also alexithymia has been associated to eating behavior [19, 40]. While some authors have observed in NT1 higher eating, shape, and weight concerns at EDE-Q compared with BMI-matched controls [21], in our sample no differences between NT1 and NT2 subjects in eating, shape, weight concerns, and BMI were detected. We did not found a positive association between eating concern and impulsivity questionnaires, probably for the small sample size.

Finally, we did not found any differences in the comparison between treated and drug-naïve patients. We cannot fully explain these findings, but we assume that the similar ESS score in the two groups may partially justify this data. Moreover, it can be assumed that drugs used in narcolepsy have no effect on non-sleep symptoms.

Several limitations in our study need to be pointed out: Firstly, the small sample size. Secondly, CSF HCRT-1 measurements were available in a minority of cases; thus, possible correlations between scores at self-reported questionnaires and HCRT-1 levels were not assessed. Thirdly, treated and drug-naïve narcoleptic patients were included, but the recruitment of both groups may increase the generalizability of the results to narcoleptic patients in real-life conditions. Further studies, with large cohorts of patients and exploring possible effects of drug treatments in NT1 and NT2 patients, are needed.

In conclusion our findings suggest that (i) NT1 exhibit higher depressive disorders and emotional dysregulation compared to healthy subjects; (ii) NT1 and NT2 patients share several similarities mainly regarding depressive symptoms, anxiety, impulsiveness, and emotion dysregulation; (iii) compared to NT2, NT1 patients exhibit higher difficulties in identifying and describing feelings maybe related to cataplexy; iv) hypnagogic hallucinations are positively correlated with emotion dysregulation; and (v) depression, anxiety, emotion dysregulation, and alexithymia appear to be correlated, supporting the hypothesis of mutual interaction of the above areas in narcolepsy.
